# Genome-wide copy number variant data for inflammatory bowel disease in a caucasian population

**DOI:** 10.1016/j.dib.2019.104203

**Published:** 2019-07-02

**Authors:** Svetlana Frenkel, Charles N. Bernstein, Yong Won Jin, Michael Sargent, Qin Kuang, Wenxin Jiang, John Wei, Bhooma Thiruvahindrapuram, Stephen W. Scherer, Pingzhao Hu

**Affiliations:** a Department of Biochemistry and Medical Genetics, The George and Fay Yee Centre for Healthcare Innovation, University of Manitoba, Winnipeg, MB, Canada; b Department of Internal Medicine, The University of Manitoba IBD Clinical and Research Centre, University of Manitoba, Winnipeg, MB, Canada; c Division of Biostatistics, Dalla Lana School of Public Health, University of Toronto, Toronto, ON, Canada; d The Centre for Applied Genomics, Genetics and Genome Biology, The Hospital for Sick Children, Toronto, ON, Canada; e Department of Molecular Genetics, University of Toronto, Toronto, ON, Canada; f Department of Electrical and Computer Engineering, University of Manitoba, Winnipeg, MB, Canada

## Abstract

Genome-wide copy-number association studies offer new opportunities to identify the mechanisms underlying complex diseases, including chronic inflammatory, psychiatric disorders and others. We have used genotyping microarrays to analyse the copy-number variants (CNVs) from 243 Caucasian individuals with Inflammatory Bowel Disease (IBD). The CNV data was obtained by using multiple quality control measures and merging the results of three different CNV detection algorithms: PennCNV, iPattern, and QuantiSNP. The final dataset contains 4,402 CNVs detected by two or three algorithms independently with high confidence. This paper provides a detailed description of the data generation and quality control steps. For further interpretation of the data presented in this article, please see the research article entitled ‘*Copy number variation-based gene set analysis reveals cytokine signalling pathways associated with psychiatric comorbidity in patients with inflammatory bowel disease*’.

**Table:** Specifications table

Subject area	*Genetics*
More specific subject area	*Copy number variant analysis*
Type of data	*Tables*
How data was acquired	*Copy number variants (CNVs) were detected from the DNA microarray-based genotypes using three independent computational algorithms*
Data format	*Filtered and analyzed*
Experimental factors	*The blood samples were collected from 243 Caucasian individuals with Inflammatory Bowel Disease (IBD) enrolled to The Manitoba IBD Cohort Study*
Experimental features	*The blood samples were genotyped using Illumina Omni2.5M-8 microarray. CNVs were detected using three independent computational algorithms: PennCNV, iPattern, and QuantiSNP*
Data source location	*Manitoba, Canada*
Data accessibility	*The list of filtered stringent CNVs was deposited to dbVar database at NCBI as the following:*https://www.ncbi.nlm.nih.gov/dbvar/studies/nstd157/
Related research article	*S. Frenkel, C.N. Bernstein, M. Sargent, W. Jiang, Q. Kuang, W. Xu, P. Hu, Copy number variation-based gene set analysis reveals cytokine signalling pathways associated with psychiatric comorbidity in patients with inflammatory bowel disease, Genomics. (2019).*https://doi.org/10.1016/j.ygeno.2019.05.001*.*

**Table:** 

**Value of the data** • The IBD CNV data set provides a valuable resource for identifying potential causal genes for IBDs and its drug targets. • It can be used as a baseline to compare and analyze the CNVs identified in other populations. • These data will be useful to researchers to investigate the contribution of CNVs to IBD and its subtypes

## 1 Data

**Fig. 1:**
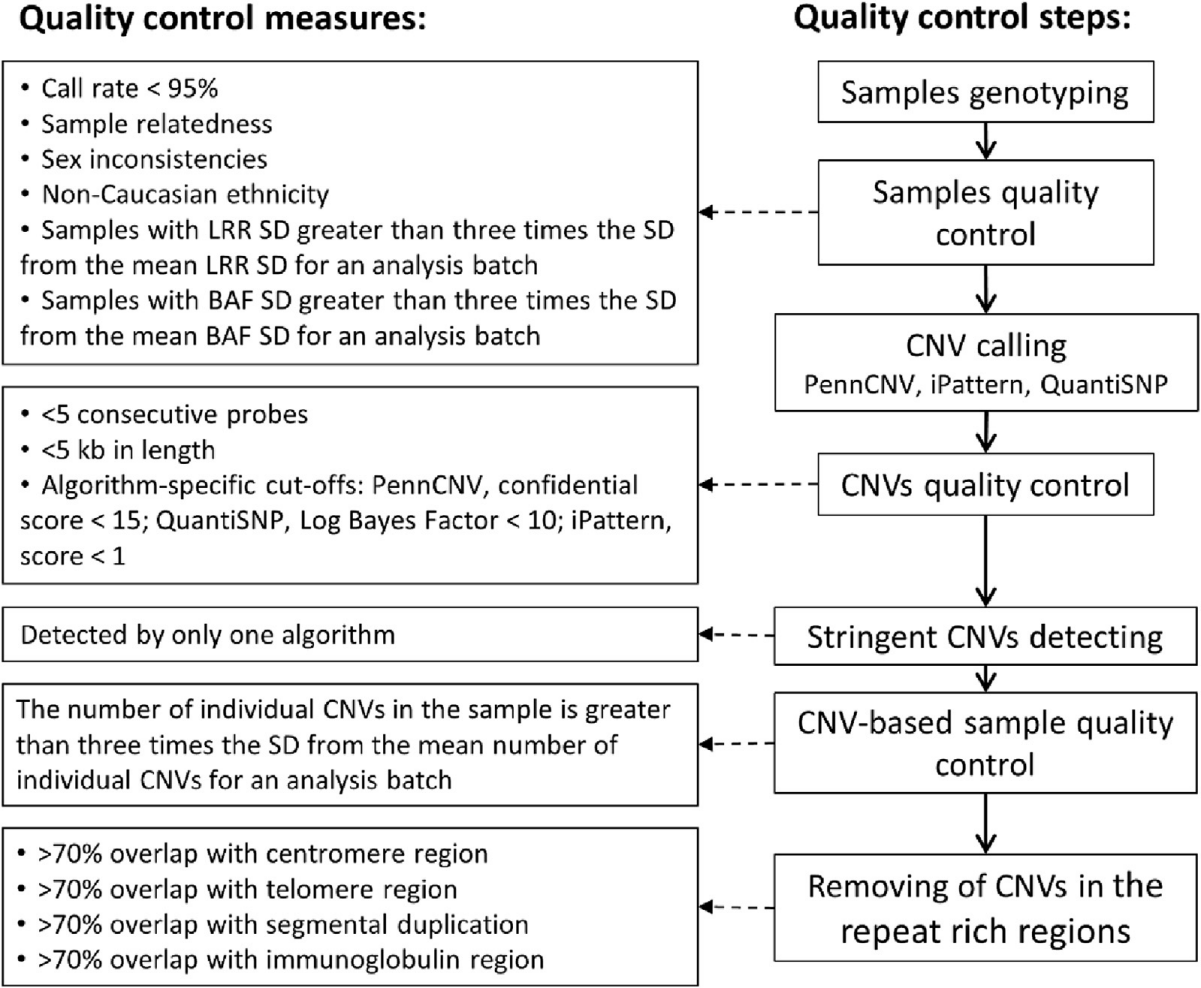
**Quality control and CNV detecting workflow.** Stringent CNV calling was conducted using variants detected by two or three calling algorithms, which were of sizes greater than 5 kb and spanned at least five array probes. SD: Standard deviation; LRR: Log R ratio; BAF: B allele frequency.

**Fig. 2:**
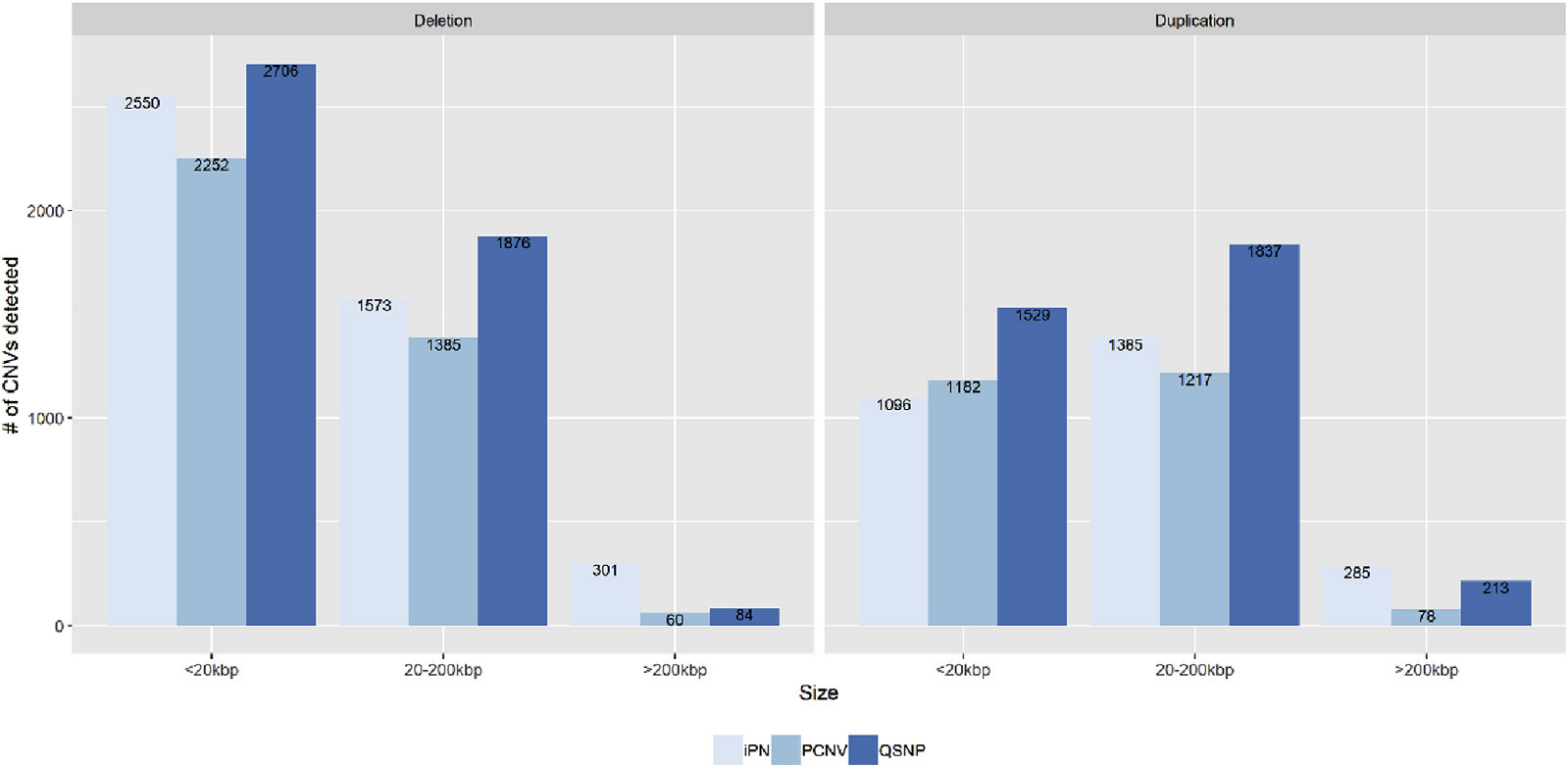
N**umber of CNVs detected by different algorithms for each size group.** Labels inside the bars indicate the exact number of CNVs detected for each category after sample and CNV quality control but before CNV merging. In the legends, iPN = iPattern; PCNV = PennCNV; and QSNP = QuantiSNP.

**Fig. 3:**
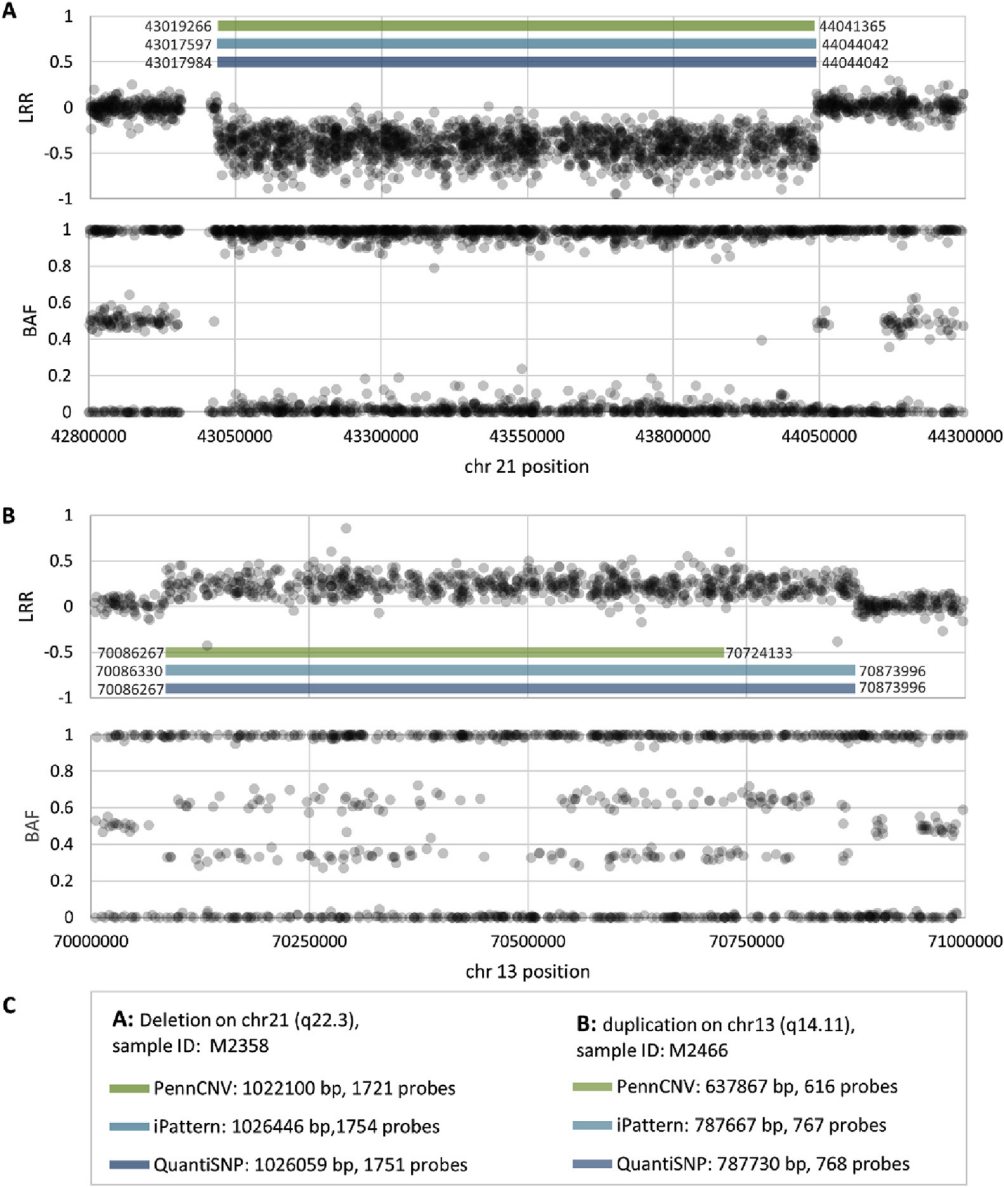
**Examples of CNV deletion and duplication detected by all three algorithms.** CNV length and number of probes for each algorithm were provided. Start and end probe positions for each algorithm were presented on the corresponding bars. Outer start and end positions were used as start and end positions of the stringent CNV. **A**: An example of deletion found in chr21q22.3; **B**: An example of duplication found in chr13q14.11; **C**: the start and end positions of the CNV detected by each of the algorithms and corresponding number of probes. LRR: Log R ratio; BAF: B allele frequency.

The presented report is a description of the CNVs identified in 243 IBD patients with Caucasian ethnicity enrolled in the Manitoba IBD Cohort Study [1]. We genotyped 269 individuals with IBD using the Illumina Omni2.5M − 8 microarray. After sample quality control and population stratification analysis, we initially selected 246 IBD patients of Caucasian ethnicity. Three different CNV detection algorithms were applied to analyze the data: PennCNV [2], iPattern [3], and QuantiSNP [4]. The detected CNVs were filtered under stringent quality control criteria for their size, probe content, and algorithm-specific quality score. The quality control workflow is presented in Fig. 1. The quality control criteria and corresponding number of disqualified samples are presented in Table 1. To obtain high-confidence calls, we removed the CNVs detected by only one of the three algorithms while the CNVs detected by two or three algorithms were merged by retaining the outer boundary [5]. Numbers of CNVs detected by different algorithms are presented in Fig. 2. The examples of merging of the results obtained by three algorithms are presented on Fig. 3. Three IBD samples with extremely large number of detected CNVs were removed, which left 243 IBD samples for the further analysis. Of the remaining data, CNVs with significant overlap with the repeat rich regions, such as centromeres and telomeres, segmental duplications, and immunoglobulin regions, were excluded [2], [6]. Table 2 contains numbers of CNVs detected by each algorithm, corresponding numbers of disqualified CNVs, CNVs qualified for merging, and removed due to overlapping with the repeat-rich regions.

**Table 1:** QC criteria and their cutoffs for sample qualification and the corresponding number of disqualified samples.

QC criterion	QC cutoff for qualification	Number of disqualified samples
Call rate	>95%	3
SD for BAF	Between −0.009 and 0.074	
SD for LRR	Between 0.042 and 0.131	2
Population outliers	European ancestry	18
Number of CNVs	<145	3

**Table 2:** **Overview of the number of CNVs after each step of quality control.** Numbers of CNVs (deletions and duplications separately, and together) detected by each of the three algorithms, number of disqualified and qualified CNVs, number of CNVs detected by each combination of algorithms, and number of CNVs removed due to overlapping with the regions of chromosomal instability.

	Duplications	Deletions	All CNVs
**Initially detected**			
PennCNV	3776	6763	10539
iPattern	3590	10199	13789
QuantiSNP	11194	18413	29607
**Disqualified CNVs in QC**			
PennCNV	1299	3066	4365
iPattern	824	5775	6599
QuantiSNP	7615	13747	21362
**Qualified CNVs for merging**			
PennCNV	2477	3697	6174
iPattern	2766	4424	7190
QuantiSNP	3579	4666	8245
**Detected CNVs by two or three algorithms**			
PennCNV, iPattern, QuantiSNP	1316	2682	3998
PennCNV, QuantiSNP	507	300	807
iPattern, QuantiSNP	330	603	933
PennCNV, iPattern	20	68	88
All stringent	2173	3653	5826
**Removed CNVs due to overlapping with regions of chromosomal instability**			
>70% overlap with segmental duplication	440	601	1041
>70% overlap with centromere, telomere or immunoglobulin region	225	228	453
Number of removed CNVs	643	781	1424
Final number of CNVs	1530	2872	4402

**Fig. 4:**
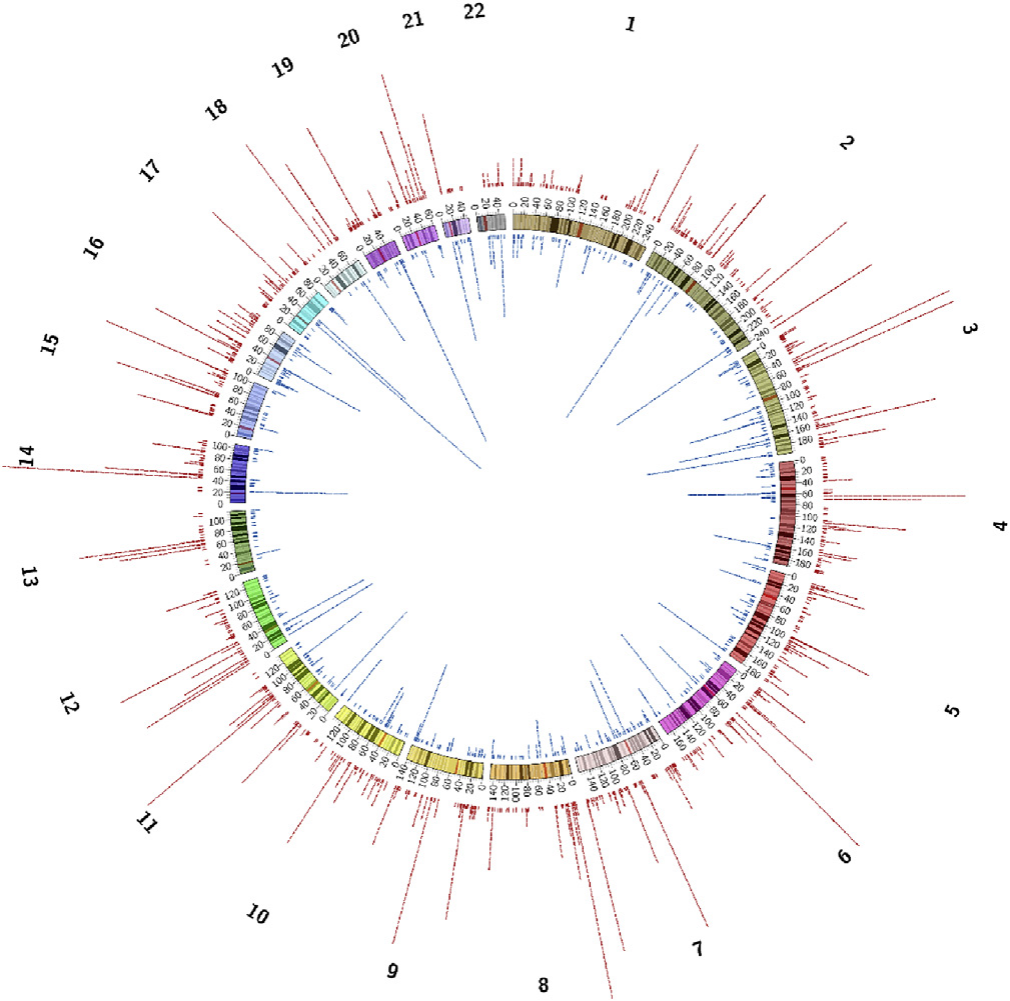
**Chromosome view of the detected CNVs.** The CNVs were presented as tiles on the corresponding genomic positions. The colour of the tile indicated the CNV type: deletions are red, duplications are blue. The height of tile stack in each genomic region corresponds to the number of CNVs; if a genomic region contains more than 30 CNVs, only 30 tiles were presented. The figure was built using the Circos [12] tool.

After the quality control and filtering, 4,402 stringent CNVs remained for the analysis; of those, 2,872 were deletions and 1,530 duplications. The chromosomal distribution of the stringent CNVs is presented on Table 3; the same data is visually presented on Fig. 4.

**Table 3:** **Chromosomal distribution of the stringent CNVs.** Number of deletions and duplications in three size categories, summary numbers of deletions and duplications and the total number of CNVs were presented for each chromosome excluding sex chromosomes. del: deletion and dupl: duplication.

Chromosome	<20 kbp, del/dupl	20–200 kbp, del/dupl	>200 kbp, del/dupl	all sizes, del/dupl	all CNV
1	79/36	38/52	4/5	121/93	214
2	126/88	50/29	20/4	196/121	317
3	141/47	79/83	3/4	223/134	357
4	110/54	53/40	1/5	164/99	263
5	100/36	56/39	3/2	159/77	236
6	84/18	98/42	2/8	184/68	252
7	138/44	47/28	17/6	202/78	280
8	137/34	45/23	2/4	184/61	245
9	111/44	30/28	2/2	143/74	217
10	72/24	29/28	3/12	104/64	168
11	125/62	15/13	2/4	142/79	221
12	149/12	20/61	0/1	169/74	243
13	54/12	37/10	0/2	91/24	115
14	53/10	105/12	2/23	160/45	205
15	122/2	10/12	0/3	132/17	149
16	63/50	41/45	9/3	113/98	211
17	53/20	16/84	2/4	71/108	179
18	80/17	12/4	0/0	92/21	113
19	18/28	59/54	3/2	80/84	164
20	57/19	36/8	1/1	94/28	122
21	6/16	19/29	1/0	26/45	71
22	11/4	8/26	3/8	22/38	60
All autosomes	1889/677	903/750	80/103	2872/1530	4402

## 2 Experimental design, materials and methods

### 2.1 Study population

Individuals were enrolled in The Manitoba IBD Cohort Study – a population-based longitudinal study of patients with IBD [7], [8]. At enrolment in the Cohort Study, participants were at least 18 years of age with a median disease duration of 4.3 years and maximal disease duration of 7 years. Participants were identified and recruited from a population-based registry, the University of Manitoba IBD Research Registry. The diagnosis of IBD was determined based on surgical, endoscopic, and histologic data. At the time of the cohort study recruitment, there were 3192 participants in the research registry. The Manitoba IBD Cohort Study was approved by the University of Manitoba Health Research Ethics Board, and participants provided written informed consent. Blood samples for genotyping were obtained from a total of 269 IBD patients.

### 2.2 Genotyping

Blood samples acquired from the 269 IBD patients in the cohort were genotyped using Illumina Infinium Omni2.5-8 microarray at The Centre for Applied Genomics (TCAG) in Toronto. Rigorous quality control (QC) procedures were performed on the resulting data. The Illumina Infinium Omni2.5-8 microarray contains a total of 2,372,784 markers for SNP and CNV analyses. Samples were processed using the manufacturer's recommended protocol; BeadChips were scanned on the Illumina BeadArray Reader using default settings. Analysis and intra-chip normalization were performed using Illumina's GenomeStudio software v.2011.1. Probes reclustering was conducted in the GenomeStudio using the project-specific samples to produce custom cluster file, which was applied to generate LogR ratios (LRR) and B Allele Frequencies (BAF) for the CNV detection.

### 2.3 Intensity quality control for CNV detection

Quality control (QC) for SNPs was performed at the individual SNP level (Table 1). Samples were excluded from the analysis if they had: i) array call rate <95%; ii) standard deviation (SD) for LRR and BAF values outside mean ± 3SD for SD of an analysis batch. Closely related samples (by identity-by-descent distance for each pair of individuals), duplicates, samples with gender mismatches (by X chromosome homozygosity rate) or Mendelian error rate >1% were excluded from the analysis batch.

### 2.4 Population stratification analysis

The reference population for population stratification analysis using multidimensional scaling (MDS) was obtained from Phase 3 data of 1000 Genomes Project [9]. Population stratification was conducted using PLINK [10] version 1.07. All ethnicity outliers were removed so that only samples of European ancestry were used in this study.

### 2.5 CNV calling algorithms

For comprehensive detection of CNVs in the IBD patients, we ran three CNV calling algorithms, namely, PennCNV [2], iPattern [3], and QuantiSNP [4]. The required data for CNV analysis, i.e. within-sample normalized fluorescence (i.e. X and Y normalized values), between-sample normalized fluorescence (i.e. Log R ratios (LRR) and B allele frequency (BAF) values) and genotypes for each sample, were exported directly from Illumina's Genomestudio software. Only autosomal probes were used in the CNV analysis. In summary, 10539, 13789 and 29607 CNVs were detected by PennCNV, iPattern and QuantiSNP, correspondingly (Table 2). We excluded the CNVs if they failed the following quality control criteria: <5 probes, <5000 bp in length and low algorithm-specific confidence score (PennCNV confidential score < 15, QuantiSNP Log Bayes Factor < 10 or iPattern score < 1). After this filtering, 6174, 7190 and 8245 CNVs were identified as high quality CNVs calls for PennCNV, iPattern and QuantiSNP, respectively. Each algorithm performed differently in calling CNVs of different sizes, with PennCNV being the most conservative in detection of CNVs, while QuantiSNP was least conservative except for large (>200 kbp) CNVs (Fig. 2).

### 2.6 CNV merging

To obtain stringent CNV calls, we merged high quality CNVs detected by at least two of the three algorithms using outer probe boundaries (Fig. 3). All CNVs detected by only one algorithm were excluded from the further analysis. As an additional step of sample QC, we excluded three samples with excessive number of stringent CNVs. We removed the samples with more than 145 CNVs (as the mean number of CNVs plus 3 SD). After CNV merging, 5826 CNVs were considered as stringent. 2173 and 3653 of the CNVs are duplications and deletions, respectively (see Table 2).

### 2.7 CNV filtering

We further excluded CNVs that: 1) overlapped the centromere (100 kbp regions before and after centromeres) or the telomere (100 kb from the ends of the chromosome); 2) had > 70% of its length overlapping a segmental duplication using the entire segmental duplication dataset downloaded from the University of California, Santa Cruz (UCSC) Genome Browser website [11]; 3) had >70% overlap with immunoglobulin region (susceptible to somatic changes) [2], [6].

The final CNV data set includes a total of 4402 CNVs, 2872 and 1530 of which were deletions and duplications, respectively (Fig. 4). The final list of stringent CNVs is available in the dbVar database at NCBI: ***nstd157***. Of all CNVs, 58.3% were smaller than 20 kbp, while 4.2% covered more than 200 kbp. Chromosomal distribution of the stringent deletions and duplications in three size categories (less than 20 kbp, 20–200 kbp and more than 200 kbp) were presented in the Table 3.
